# The RNA Polymerase Dictates ORF1 Requirement and Timing of LINE and SINE Retrotransposition

**DOI:** 10.1371/journal.pgen.1000458

**Published:** 2009-04-24

**Authors:** Emily N. Kroutter, Victoria P. Belancio, Bradley J. Wagstaff, Astrid M. Roy-Engel

**Affiliations:** 1 Tulane Cancer Center SL-66, Tulane University Health Sciences Center, New Orleans, Louisiana, United States of America; 2 Department of Epidemiology, Tulane School of Public Health and Tropical Medicine, New Orleans, Louisiana, United States of America; 3 Department of Structural and Cellular Biology, Tulane School of Medicine, New Orleans, Louisiana, United States of America; 4 Tulane Center for Aging, Tulane School of Medicine, New Orleans, Louisiana, United States of America; Fred Hutchinson Cancer Research Center, United States of America

## Abstract

Mobile elements comprise close to one half of the mass of the human genome. Only LINE-1 (L1), an autonomous non-Long Terminal Repeat (LTR) retrotransposon, and its non-autonomous partners—such as the retropseudogenes, SVA, and the SINE, *Alu*—are currently active human retroelements. Experimental evidence shows that *Alu* retrotransposition depends on L1 ORF2 protein, which has led to the presumption that LINEs and SINEs share the same basic insertional mechanism. Our data demonstrate clear differences in the time required to generate insertions between marked *Alu* and L1 elements. In our tissue culture system, the process of L1 insertion requires close to 48 hours. In contrast to the RNA pol II-driven L1, we find that pol III transcribed elements (*Alu*, the rodent SINE B2, and the 7SL, U6 and hY sequences) can generate inserts within 24 hours or less. Our analyses demonstrate that the observed retrotransposition timing does not dictate insertion rate and is independent of the type of reporter cassette utilized. The additional time requirement by L1 cannot be directly attributed to differences in transcription, transcript length, splicing processes, ORF2 protein production, or the ability of functional ORF2p to reach the nucleus. However, the insertion rate of a marked *Alu* transcript drastically drops when driven by an RNA pol II promoter (CMV) and the retrotransposition timing parallels that of L1. Furthermore, the “pol II *Alu* transcript” behaves like the processed pseudogenes in our retrotransposition assay, requiring supplementation with L1 ORF1p in addition to ORF2p. We postulate that the observed differences in retrotransposition kinetics of these elements are dictated by the type of RNA polymerase generating the transcript. We present a model that highlights the critical differences of LINE and SINE transcripts that likely define their retrotransposition timing.

## Author Summary

SINE retroelement amplification has been extremely successful in the human genome. Although these non-autonomous elements parasitize factors from LINEs, both the human *Alu* and the cumulative rodent SINEs have generated over one million copies in their respective hosts. *Alu*-induced mutagenesis is responsible for the majority of the documented instances of human retroelement insertion-induced disease. Our data indicate that SINEs require a shorter period of time to complete insertion than L1s, possibly contributing to the ability of *Alu* elements to effectively parasitize L1 components. We demonstrate that RNA polymerase changes the timing *Alu* requires to complete retrotransposition and creates the need for the L1 ORF1protein in addition to ORF2p. We postulate that the way cells manage pol III and pol II (mRNA) transcripts affects the timing of a transcript going through the retrotransposition pathway. We propose a model that highlights some of the critical differences of LINE and SINE transcripts that likely play a crucial role in their retrotransposition process.

## Introduction

Mobile elements have constantly assaulted genomes, shaping and molding their structure and organization. In particular, mobile elements have flourished in mammals generating between 40–50% of their genomic sequence [1]–[3]. About one third of the human genome can be attributed directly or indirectly to the activity of the non-LTR retroelements also referred to as LINEs (Long INterspersed Elements). LINE-1 (L1) and its non-autonomous partners *Alu*, SVA, and retropseudogenes continue to amplify in the human genome. L1 and the SINE (Short INterspersed Element), *Alu*, are by far the most numerous, adding up to 1.5 million copies [1]. Although *Alu* mobilization depends on L1 proteins [4], they outnumber L1 inserts by 2 to 1. Similarly, the sum of the total copies of all rodent SINEs outnumber L1 copies about 2 to 1 [2],[3]. *Alu* and the rodent SINE inserts have been more successful than other non-autonomous retroelements, such as the retropseudogenes [5]. Size and sequence composition differences between SINEs and LINEs may allow the mammalian genome to better tolerate SINE insertions, reviewed in [6]. Negative selection has clearly played a role in reducing L1 copy number through ectopic recombination and elimination of many full length and nearly full length L1 inserts [7]. However, processes other than negative selection must influence the observed differences. The updated reports of diseases caused by *de novo* inserts (where little, or no, selection has occurred) show that *Alu* inserts outnumber those of L1 by about 2 to 1 [6],[8].

Tissue culture assay systems indicate that L1 retrotransposition rates are consistently higher than those observed for SINEs [4],[9]. This is possibly a reflection of the strong cis-preference contained by L1 [10],[11], while *Alu* must compete for L1 proteins in *trans*. How is it that *Alu* with a lower retrotransposition rate than L1, contributes more *de novo* disease cases? It is likely that multiple factors are involved, such as the ability to bind SRP9/14 [12],[13].

**Figure 1:**
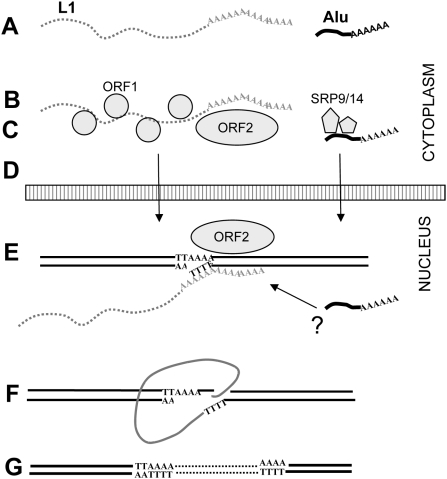
Model of the Retroelement Retrotransposition Cycle. A–G represent individual steps in the retrotransposition cycle: A. The first step requires the transcription of the RNA, processing and export to cytoplasm. B.and C. L1 protein translation needs to occur and both SINE and LINE RNPs form in the cytoplasm. L1 ORF1 and ORF2 proteins are represented by small and large circles, respectively. The SRP9 and SRP14 proteins are represented by pentagons. D. The RNA and proteins reach the nucleus in an unknown manner. In the nucleus: E. To prepare for insertion, the DNA is cleaved by the L1 ORF2p endonuclease. The L1 endonuclease cleaves at AT-rich sequences with the consensus 5′-TTAAAA-3′/3′-AA_↑_TTTT-5′. At this stage the “A-tail” of the L1 or *Alu* transcript is thought to interact with the cleaved DNA. It is proposed that reverse transcription occurs through a process referred to as target primed reverse transcription (TPRT). The L1 ORF2p reverse transcriptase generates the first strand of DNA. It is unknown whether or not SINE RNA can be involved in a template switch or compete for L1 factors at this step (indicated by the “?”). F. Completion of the retrotransposition requires second-strand synthesis, a second nick caused by an unknown source, and ligation of the 3′ end of the cDNA to the genome. At least some of these steps could involve endogenous cellular activities. DNA repair processes are likely to be involved in the final steps. G. The end product results in the generation of an insert with the hallmark direct repeats.

Retroelements are mobile elements that amplify through an RNA intermediate in a process known as retrotransposition [14]. There are limited data on the details of the mechanism of LINE retrotransposition, and even less for SINE retrotransposition. The process begins with the generation of RNA (Figure 1A). Active L1 elements express two proteins from a bicistronic mRNA: ORF1p[15] and ORF2p (Figure 1B and C). Both L1 proteins are needed for L1 retrotransposition [16]. In contrast to L1, ORF2p expression is sufficient for SINE retrotransposition [4],[9],[17], while ORF1p may enhance the process [17]. ORF1p possesses nucleic acid chaperone activity [18],[19], an essential property for L1 retrotransposition [19],[20]. ORF2p is a multifunctional protein with endonuclease and reverse transcriptase activities [21],[22]. Both proteins are proposed to interact in *cis* [10],[11] with the L1 RNA to form a cytoplasmic RNP complex interacting with polyribosomes [20],[23]. SINE RNA is predominantly found in the cytoplasm as an RNP complex [12],[24],[25] (Figure 1C) and uses L1 protein(s) in *trans* for its mobilization. The endonuclease of the L1 ORF2p generates the first nick within the L1 endonuclease recognition sequence generating single stranded DNA that primes the reverse transcription [22],[26]. Both L1 and *Alu* are proposed to undergo integration through a target-primed reverse transcription (TPRT) reaction [27].

To generate a new insertion, L1 and SINE elements must return to the nucleus either together or independently (Figure 1D). Reported data suggest that retrotransposition-competent L1 RNPs may transit through the nucleolus [28]. The 3′ poly-A stretch or “A-tail” of LINEs, SINEs and processed pseudogenes is required for the priming of reverse transcription (Figure 1E) [4],[29]. Unlike the post-transcriptionally generated A-tail of pol II RNAs (mRNA), SINE A-tails are included within their sequence and play an important role in SINE retrotransposition [30],[31]. The details of the final integration and ligation of the L1 or *Alu* inserts into the host DNA remain unclear. Recent reports indicate that cellular factors, such as DNA repair enzymes, may aid in the L1 retrotransposition process [32],[33]. The final inserted sequence is typically flanked by direct repeats (Figure 1G). Non-autonomous retrotransposed inserts, such as *Alu*, SVA, hYs and retropseudogenes share these hallmarks with L1 inserts, strongly suggesting that these elements use the L1 ORF2p endonuclease generated nick for their integration [34]–[36].

To date, all known SINEs are ancestrally derived from RNA pol III transcribed RNA genes, reviewed in [37]. The vast majority are derived from different tRNA genes and only two (*Alu* and the rodent B1) originated from the 7SL RNA gene, a component of the signal recognition particle (SRP) [38]. Other examples of pol III transcribed repeats include the four hY genes (hY1, hY3, hY4 and hY5) that likely contributed directly or indirectly to the generation of almost 1000 copies in the human genome by retrotransposition [36],[39]. In contrast to SINEs, an internal RNA pol II promoter drives LINE transcription with the unusual ability to start transcription upstream of its location. Like other pol II RNAs, L1 transcription is regulated by different mechanisms, including promoter methylation [40], transcriptional attenuation due to A-richness [41], premature polyadenylation [42], and the generation of different splice variants [6]. Additional studies suggest that at least some portion of the L1 mRNAs are capped [43] and that the capping enhances L1 translation [44].

Previously, an L1 element tagged with a green fluorescent protein (EGFP) retrotransposition cassette was used to detect L1 retrotransposition “near real time” [45]. The earliest detection of an L1 retrotransposition event was 48 h post-transfection. In this manuscript, we evaluate the timing of retrotransposition (defined as the time required for a retroelement from the initial transcription step to complete an insertion) of tagged Alu and L1. We demonstrate that *Alu* elements only require about half of the amount of time as L1 to generate an insert. Our data demonstrate that the type of RNA polymerase dictates the retrotransposition timing, but does not determine the retrotransposition rate (defined as the number of inserts a given element can generate, *i.e.* the “efficiency” of an element). After evaluating several potential time limiting steps, we show that the RNA polymerase type is an important early factor contributing to the divergent retrotransposition kinetics between LINEs and SINEs.

## Results

### The Use of an HIV Reverse Transcriptase Inhibitor d4t as a Suppressor of L1 and *Alu* Retrotransposition in Culture

**Figure 2:**
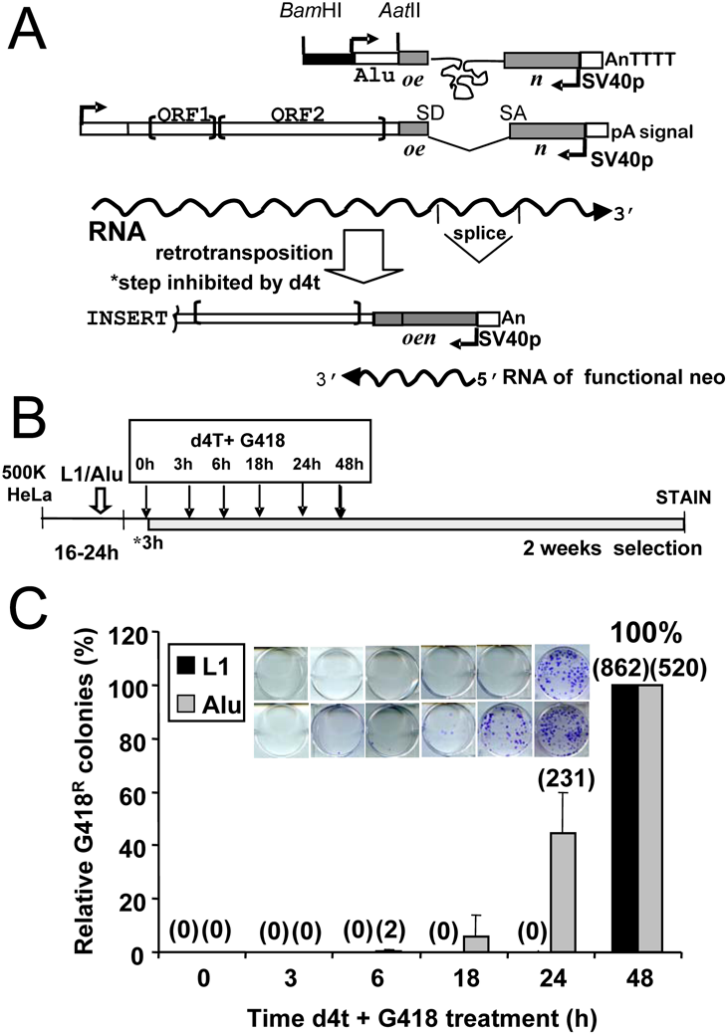
*Alu* and L1 Exhibit Different Retrotransposition Kinetics. A. Assay design. A schematic of the constructs used for the L1 and *Alu* tissue culture assay are shown on the top. RNA transcription is driven by a CMV promoter for the L1 construct or the internal pol III Alu promoter. The restriction sites used in the construction of the other pol III driven vectors are shown. The L1 construct contains a full-length retrocompetent L1 element with its ORF1 and ORF2. The L1 vector is tagged with the *mneoI* indicator cassette containing an inverted neomycin resistance gene (*neo*, light gray box) disrupted by an intron [16]. The *Alu* vector contains a *neo* ^TET^ cassette with a *tetrahymena* self-splicing intron interrupting the *neo* gene [4]. In both constructs, the introns will only splice out from a transcript generated by the L1 or *Alu* promoter. The spliced RNA is reverse transcribed, followed by integration of the cDNA into the genome. The new insert contains a functional neomycin gene. G418 resistance will be obtained only if retrotransposition occurs. B. Schematic of treatment timeline. HeLa cells were seeded and transfected the next day with the appropriate constructs. After the three hour incubation with the transfection cocktail (3h*) the first set of cells was treated with d4t and G418 containing media (0 h). Note that at this time point the plasmid DNA has already been in contact with the cells for 3 h. The second set of cells was treated after 3 hours (3 h), and so forth until completing all the time points (shown as arrows above). Cells were stained after 2 weeks of growth under selection. C. *Alu* inserts are detected at 24 h, while L1 requires at least 48 hours to generate inserts. HeLa cells were transiently transfected with L1*mneo* (black bar) or AluYa5*neo* ^TET^+ORF2p expression vector (gray bar) and d4t plus G418 treatment started 3, 6, 18, 24, and 48 h post-transfection (x axis). Inset shows representative G418^R^ foci results of the retrotransposition assay. Bars represent the relative % mean G418^R^ colonies±standard deviation shown as error bars for each construct. The 48 h data were used to define 100%. The mean of the observed G418 resistant colonies is shown in parentheses above each column.

Reverse transcriptase (RT) domains of multiple sources can be grouped into a family of shared sequence homology [NCBI cdd pfam00078.12] [46], including the RT of the human immunodeficiency virus and L1 ORF2 protein. Endogenous RT activity is inhibited by two antiretroviral agents nevirapine and efavirenz [47]. L1 retrotransposition in a culture assay system can be suppressed by the addition of a variety of HIV RT inhibitors [48],[49]. This system utilizes a tagged vector designed to allow expression of the reporter gene only when the retroelement goes through its reverse transcriptase-dependent amplification process (Figure 2A). Therefore, only the newly inserted element will express the reporter gene (*e.g.* neo).

Using the established L1 and *Alu* retrotransposition tissue culture assays [4],[16], we evaluated the dose of, 2′,3′-didehydro-3′-deoxy-thymidine (d4t) required to abolish retrotransposition of L1 and L1 ORF2p driven *Alu* without adversely affecting cell growth and viability. Treatment of transiently transfected HeLa cells showed that both L1 and *Alu* activities presented a d4t activity inhibitory concentration 50 (IC_50_) of about 2 µM (Figure S1). For our subsequent experiments we utilized d4t treatments at 50 µM (25 fold the IC_50_) to inhibit SINE and LINE retrotransposition in tissue culture. We selected this dose for its efficient inhibition of retrotransposition and lack of observed negative effects, determined by colony formation of an unrelated plasmid that expresses a functional neomycin resistance gene and integrates into genomic DNA by random integration rather than by an L1-dependent mechanism (data not shown).

### Generation of L1NE-1 and *Alu* Insertions Have Different Time Requirements

We took advantage of the d4t inhibition to determine L1 and *Alu* retrotransposition kinetics in cultured cells. By treating cells with d4t at different time points after the transient transfection with the vectors expressing the tagged L1 or *Alu* plus ORF2p, we specifically inhibited the retrotransposition process at designated time periods (shown in Figure 2B). Any detected L1 or *Alu* inserts are presumed to have completed the insertion process prior to the addition of the d4t, as inhibition of ORF2p RT activity would prevent the generation of the cDNA. Using this approach, we show that L1 inserts are not detected in cultured cells during the first 24 h post-transfection (Figure 2C). Similar results were previously observed using a green fluorescent protein (EGFP)-tagged L1 element [45],[50]. The earliest detection of L1 inserts occurred at 32 h post-transfection (Figure S2). In contrast, we can easily detect *Alu* inserts 24 h and sometimes as early as 18 h post-transfection (Figure 2C).

### Availability of L1 RNA Is Not a Limiting Factor

**Figure 3:**
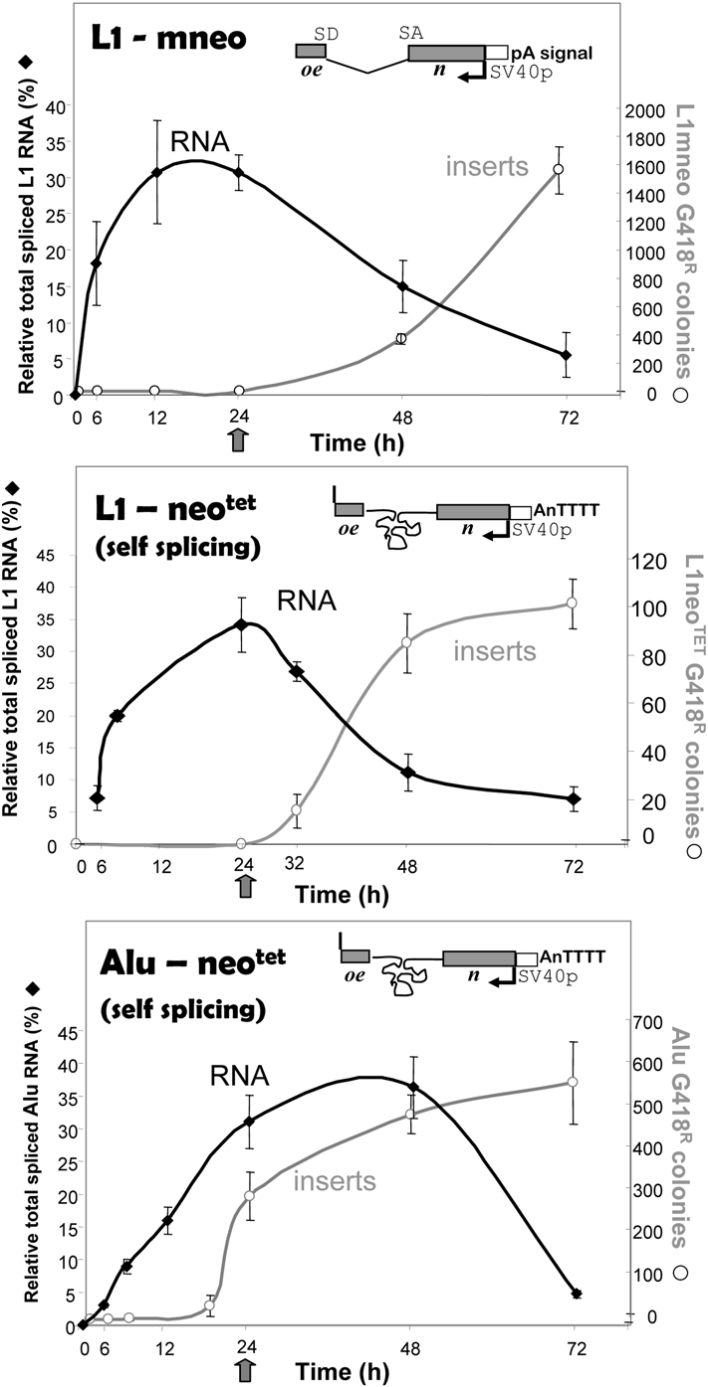
Availability of Spliced L1 RNA Is Not a Limiting Factor. Transcription and retrotransposition kinetics were evaluated for the different constructs. HeLa cells were transiently transfected with L1*mneo*, AluYa5*neo* ^TET^+ORF2p or L1*neo* ^TET^ (with the same self-splicing *neo* cassette used for the *Alu* construct). Cells were either harvested for RNA quantitation (left y axis, black square) or treated with d4t plus G418 treatment for colony quantitation (right y axis, gray circles) at the indicated time points post-transfection (x axis). RNA was quantitated relative to β-actin as control (details in Materials and Methods). Note that the colony numbers reflect the actual cumulative inserts that occurred from transfection to the d4t treatment time point. The data demonstrate that all constructs generate the spliced tagged transcripts at early time points in a similar manner; however the observed inserts between *Alu* and L1 differ at 24 h.

Generation of an RNA transcript is an essential first step of the retrotransposition cycle (Figure 1A). Besides serving as a template for protein translation, L1 RNA acts as the insertion template during retrotransposition. Thus, transcriptional limitations or variations can directly impact retrotransposition of L1 elements as well as other retroelements. Previous studies demonstrate that L1 elements generate low amounts of full-length transcripts due to premature polyadenylation [42], transcriptional inefficiency due to A-richness [41], and multiple splicing events [6]. In all these studies, a decrease in the amount of L1 mRNA contributed to reduced retrotransposition and, conversely, the rate increased with higher amounts of full-length L1 RNA [42],[51],[52]. To determine whether L1 RNA transcription and processing contributes to the observed timing difference between L1 and *Alu* inserts, we performed a time course to evaluate the generation of the spliced RNA product in cells transiently transfected with L1*mneo*, *Alu*Ya5*neo* ^TET^, and L1*neo* ^TET^ (Figure 3). Because the *Alu* construct is driven by RNA polymerase III, its tag (*neo* ^TET^) contains a self splicing intron disrupting the neomycin gene. Therefore, we included an additional L1 construct that contains the exact same self splicing (*neo* ^TET^) tag present in the *Alu* vector to control for any potential variations introduced by splicing dynamics. Full-length spliced and unspliced transcripts from *Alu* and both L1 constructs could be detected as early as 3 hours post-transfection (northern blots shown in Figure S3). The *mneo* and *neo* ^TET^ tagged L1 constructs exhibited similar kinetics for the spliced transcript (only RNA that will generate G418^R^ colonies when retrotransposed), peaking by 24 h and declining by 72 hours (Figure 3). Splicing efficiency of the RNA produced by different expression vectors was evaluated (Table S1). Equivalent splicing efficiency was observed for the L1 and *Alu* transcripts sharing the same neomycin cassette (*neo* ^TET^ or *mneo*). *Alu*-tag transcripts were only detected in the cytoplasmic fraction at any of the time points evaluated (data not shown), consistent with what has been previously reported for the authentic *Alu* “untagged” RNA [53]. Despite early L1 mRNA availability, no L1 inserts were observed at the 24 h time point. Spliced *Alu* transcripts peak around 48 h, declining by 72 h, much like L1 mRNA (Figure 3). However, in contrast to L1, numerous *Alu* inserts are readily detectable by 24 h. These results demonstrate that the full-length properly spliced L1 RNA is generated in the same time period as the *Alu* RNA. Thus, it is unlikely that RNA transcription or variation in the type of splicing within the neo cassette account for the observed time difference between the generation of *Alu* and L1 inserts.

Another difference between the *Alu* and L1 elements involves the length of the transcript, which could alter the time required by the reverse transcriptase to generate a full-length cDNA. In this assay system full length inserts are not required to generate a G418^R^ colony. In both *Alu* and L1 assays, inserts are detected with the retrotransposition of the minimal unit of a functional neomycin gene, which is identical in length in both transcripts once the intron is removed. Therefore, the timing differences observed between these two elements should be independent of the transcript length.

### L1 ORF2p Activity Can Be Detected within 24 Hours

We next assessed whether the delay reflects the time required for translation of the L1 proteins and the ability to reach the nucleus (Figure 1B–E). ORF2 protein has been notoriously difficult to observe by conventional techniques, such as western blot analysis [28]. As an alternative, the ORF2p activities can be evaluated.

**Figure 4:**
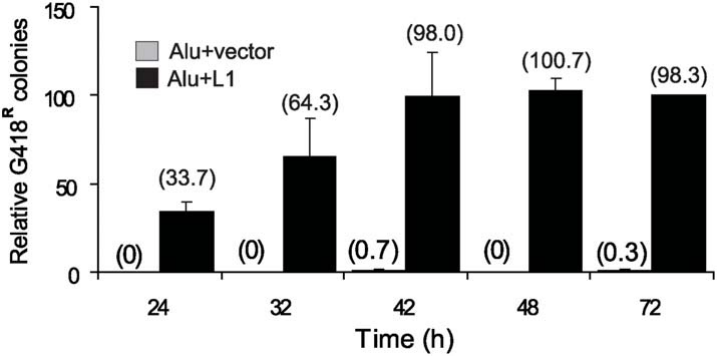
Detection of L1 ORF2p *Trans*-mobilization Activity at 24 h. HeLa cells were transiently transfected with AluYa5*neo* ^TET^ plus L1 no tag or empty vector (control). The d4t and G418 treatment was started at 24, 32, 36, 48 and 72 h post-transfection (x axis). Bars represent the relative % mean G418^R^ colonies±standard deviation shown as error bars for each construct. The 72 h data were used to define 100%. The mean of the observed G418 resistant colonies is shown in parentheses above each column. Note that for the control only 2 and 1 colonies were observed at 42 and 72 hours, respectively. The data demonstrate that functional ORF2p generated by the L1 no tag “wildtype” vector must reach the nucleus by 24 h for *Alu* retrotransposition to occur.

Because *Alu* elements require ORF2p for retrotransposition, evaluation of *Alu* retrotransposition serves as an alternate method to detect ORF2p activity. Therefore, we exploited the *trans*-complementation assay to monitor the ability of L1 to *trans*-mobilize *Alu*, using *Alu*Ya5*neo* ^TET^ as a reporter construct. We determined the *Alu* insertion kinetics in cells cotransfected with the *Alu*Ya5*neo* ^TET^ plus the L1 no tag vector. Multiple *Alu* inserts were detected as early as 24 h post-transfection (Figure 4), corroborating the availability of the ORF2p expressed from the L1 vector in the nucleus by 24 h. Equivalent results were observed when using a blasticidin tagged L1 to drive *Alu* retrotransposition (data not shown). Under our experimental conditions, endogenous L1 present in HeLa cells does not significantly contribute to the generation of the G418^R^ colonies as the *Alu* vector was unable to generate any inserts without L1 supplementation at 24, 32 and 48 h post-transfection (vector control, Figure 4). A few solitary colonies (2 and 1) were observed at the 42 and 72 h time points. This observation clearly demonstrates that a full-length L1 vector generates enough ORF2p to reach the nucleus within 24 h and to mobilize a tagged *Alu* element in our assay system. Our observations are in agreement with previously published data demonstrating that cells transiently transfected with L1 exhibit extensive double strand breaks at 24 h post-transfection [33]. The observed DNA breaks are dependent on the endonuclease activity of the L1 ORF2p. Our data strongly suggest that translation and nuclear localization of ORF2p is unlikely to be the main limiting step for the observed differences between the L1 and *Alu* time requirements.

In addition, pre-transfection of high amounts of ORF2p or any of the L1 factors (proteins and/or RNPs) in *trans* did not alter L1 retrotransposition timing (Figure S4). This is not surprising considering that L1 RNA exhibits a strong *cis*-preference for its own translated proteins for retrotransposition [10],[11]. Pre-transfection with ORF2p showed a few more *Alu* inserts at early time points (data not shown). However, this slight increase was not statistically significant (Student's paired t-test, p = 0.297).

### The RNA Polymerase Dictates SINE and LINE Retrotransposition Timing

**Figure 5:**
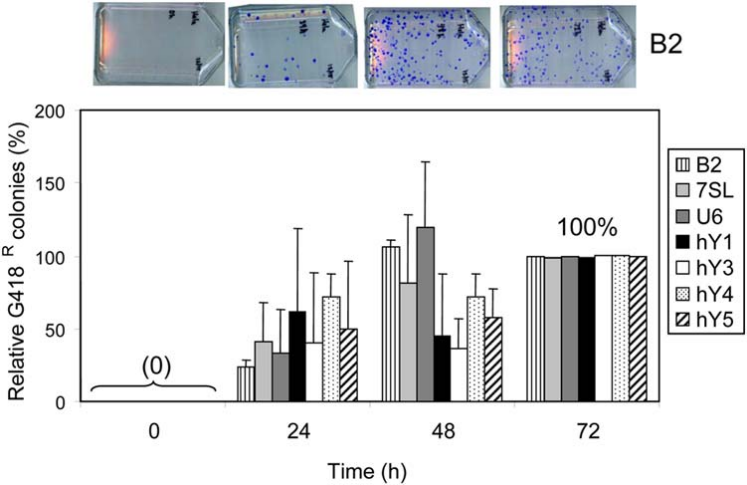
RNA Pol III Transcripts Share Similar Retrotransposition Kinetics. Other pol III genes known to generate retrotransposed copies parallel *Alu* kinetics by generating inserts within 24 h. HeLa cells were transiently transfected with the ORF2p expression vector plus the tagged vector of the rodent SINE B2 (vertical lines), and the 7SL (light gray), U6 (dark gray), hY1 (black), hY3 (white), hY4 (dotted) or hY5 (slanted-lines) RNA genes. The above Inset shows representative G418^R^ foci results of the B2 retrotransposition assay. Cells were treated with d4t+G418 at 0, 24, 48 and 72 h post-transfection. The 72 h data were used to define 100%. Bars represent the % relative mean G418^R^ colonies±standard deviation shown as error bars for each construct (n = 3).

Transcripts generated from RNA polymerase II and III promoters differ in their capping, 3′ end processing, folding structures, post-transcriptional processing, interaction with translation factors and degradation pathways, reviewed in [54]–[56]. In addition, these two transcriptional complexes can be observed in different spatial locations in the nucleus indicating discrete transcriptional sites [57],[58]. To evaluate the timing of retrotransposition of other pol III-driven genes we generated “tagged” versions of 6 human genes (7SL, U6, hY1, hY3, hY4 and hY5) by cloning the genes with at least 300 bp of their upstream enhancer sequence 5′ of the *neo* ^TET^ cassette (details in materials and methods). Although the “functional” genes are not SINEs *per se*, we selected these as examples of pol III-driven genes. The human genome contains multiple examples of retrotransposed copies with sequence homology to these genes [36],[59]. Thus, these serve as our best examples of other human pol III-driven constructs. We also included in our analysis the pol III-driven B2 element as a known active rodent SINE [9],[60]. In our d4t-assay system, all tagged pol III-driven elements generated inserts by 24 h post-transfection when supplemented with just L1 ORF2p (Figure 5).

**Figure 6:**
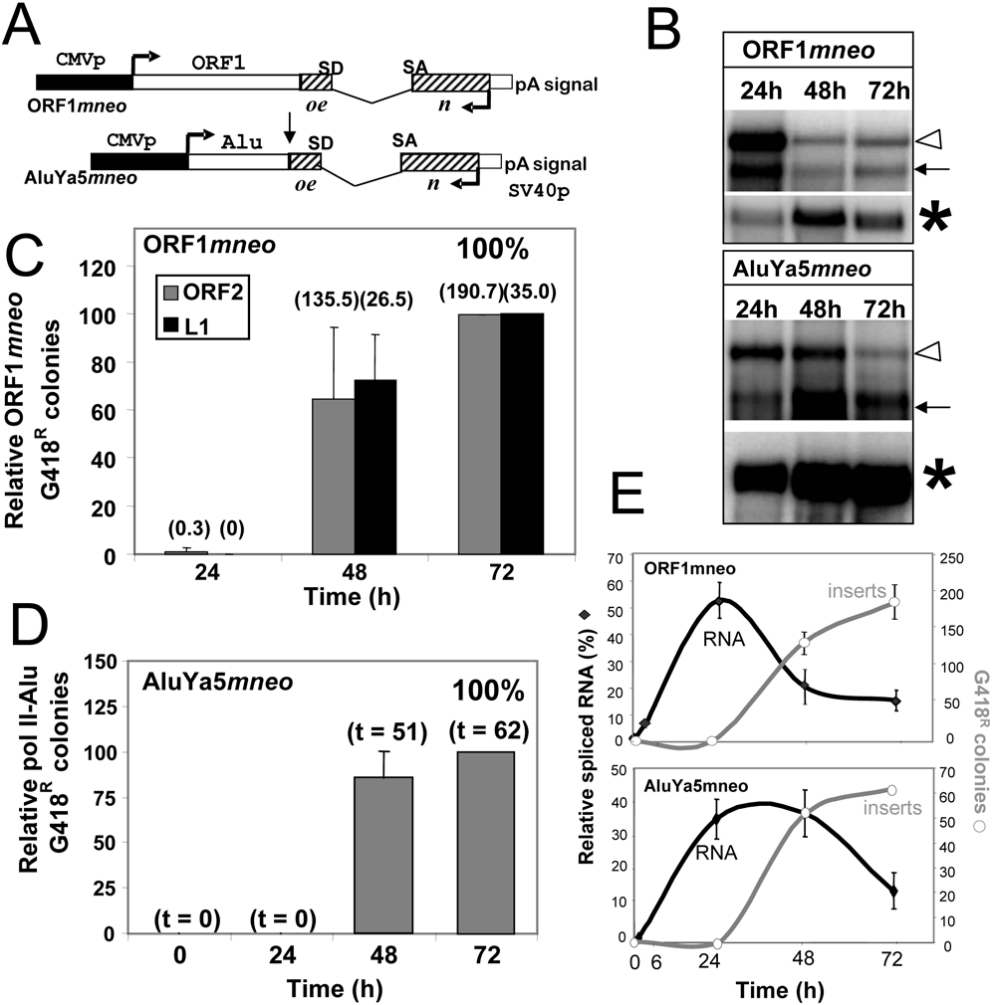
The RNA Polymerase Dictates the Retrotransposition Kinetics of *Alu*. A. Schematic of pol II-driven ORF1 and *Alu* vectors. The ORF1mneo construct was selected as a representative of retropseudogene activity. The constructs use the CMV promoter (CMVp, black box) to generate pol II transcripts. The full *mneoI* indicator cassette from the L1 vector, consisting of the neomycin interrupted by an inverted intron (hatched box), its SV40 promoter (SV40p) and complete polyadenylation signal (pA signal) is located downstream of the L1 ORF1 (ORF1*mneo*) or a consensus *Alu*Ya5 (AluYa5*mneo* “pol II *Alu*”) (arrow indicates where the *Alu* “normal A-tail” would have been located). B. Spliced RNA pol II generated transcripts of tagged ORF1 and *Alu* are readily available by 24 hours. Poly-A selected RNA extracts from different post-transfection time points (24, 48 and 72 h) were evaluated by Northern blot analysis using an RNA strand specific probe to the neomycin resistance gene. The unspliced (open arrowhead) and spliced (black arrow) transcripts from the pol II-vectors AluYa5*mneo* and ORF1*mneo* are shown. β-actin is indicated by an *. C. The tagged ORF1 transcript mimics tagged L1 insertion kinetics. Retrotransposition assays were performed using the ORF1*mneo* vector supplemented with an L1 (black) or ORF2p expression (gray) vector. Cells were treated with d4t plus G418 at 24 and 48 h post-transfection. Bars represent the relative % mean G418^R^ colonies±standard deviation shown as error bars for each construct (n = 3). The 72 h data were used to define 100%. The mean of the observed G418 resistant colonies is shown in parentheses above each column. Only one colony (1) was observed at the 24 h time point. D. Transcription from a pol II promoter alters the retrotransposition requirements of a tagged *Alu* element. The retrotransposition capability of the pol II-driven *Alu* (AluYa5*mneo*) supplemented with ORF1p and ORF2p expression vectors was evaluated in HeLa cells. Cells were treated with d4t plus G418 at 24 and 48 h post-transfection. The 72 h data were used to define 100%. Bars represent the relative % mean G418^R^ colonies±standard deviation shown as error bars for each construct (n = 6). The total number of G418 resistant colonies for all experiments combined is shown in parentheses indicated by a “t”. No colonies were ever observed at the 24 h time point. E. Transcription and retrotransposition kinetics of pol II driven ORF1 and *Alu*. HeLa cells were transiently transfected with ORF1*mneo* (top panel) or AluYa5*mneo* (lower panel) and either harvested for RNA quantitation (left y axis, black square) or treated with d4t plus G418 treatment for colony quantitation (right y axis, gray circles) at the indicated time points post-transfection (x axis). RNA was quantitated relative to β-actin as control. The data demonstrate that the generation of spliced pol II and pol III Alu transcripts are equivalent; however pol II Alu inserts are not detected at 24 h.

To better understand the RNA polymerase influence on retrotransposition, we also evaluated the time requirement of two pol II-driven (CMV) constructs: ORF1*mneo* and pol II *Alu* (Figure 6A). We selected ORF1*mneo* because it generates a transcript of L1 ORF1, which has previously been used to reflect retropseudogene activity [10]. The ORF1*mneo* vector can retrotranspose when a source of ORF2p is supplied in *trans* [10]. The pol II *Alu* (p^CMV^Ya5*mneo*) contains an *Alu* tagged with the “*mneo*” cassette from the L1-tagged construct [61], which contains pol III terminators (4 Ts) that would generate truncated transcripts if the internal pol III A and B boxes in the *Alu* sequence are used for transcription. The “normal A-tail” at the end of the *Alu* sequence and 5′ of the neo cassette (Figure 6A) was not included in order to prevent potential internal priming for TPRT in the cDNA extension step (Figure 1E), which would circumvent inclusion of the neo reporter gene in the retrotransposed copy. Thus, only the *Alu* body sequence was utilized in the construct. Just like the L1 construct, the A-tail used in the TPRT step is generated from the transcript polyadenylation by the RNA polymerase II from the SV40pA signal at the 3′ end of the neo cassette (Figure 6A). Spliced and unspliced transcripts were detected from both constructs by 24 h (Figure 6B). The tagged ORF1p transcript driven by an ORF2p generated one single insert at 24 hours (Figure 6C), while the total number of colonies generated were 136 and 226 for 48 h and 72 h respectively. It is possible that the endogenous L1 expression in HeLa cells [6] affected the timing. However, our data on *Alu* retrotransposition indicates that effects from endogenous L1 expression under our experimental conditions are negligible (Figure 4). Most likely, the single G418^R^ colony observed at 24 hours is due to a rare event that escaped d4t inhibition. A quantitative time course evaluation of the spliced RNA product in cells transiently transfected with ORF1mneo and AluYa5*mneo* further indicates that the availability of spliced product is not limiting retrotransposition timing (Figure 6E).

No pol II-generated *Alu* inserts were ever observed when supplemented with ORF2p under any conditions tested, representing a rate of less than 1×10^6^ cells/µg of plasmid. However, retrotransposition of the pol II-driven *Alu* transcript occurred when it was supplemented with both ORF2p and ORF1p expression plasmid (Figure 6D). Under these conditions, G418^R^ colonies were observed at 48 h post-transfection, much like L1 and retropseudogene behavior. No colonies were ever observed at the 24 h time point in 5 independent experiments using triplicates for each time point. Swapping the RNA pol III for an RNA pol II promoter changed the retrotransposition requirements of the tagged *Alu* to reflect those observed for pseudogenes and LINEs.

### The Timing of Retrotransposition Does Not Predict Insertion Rate

**Figure 7:**
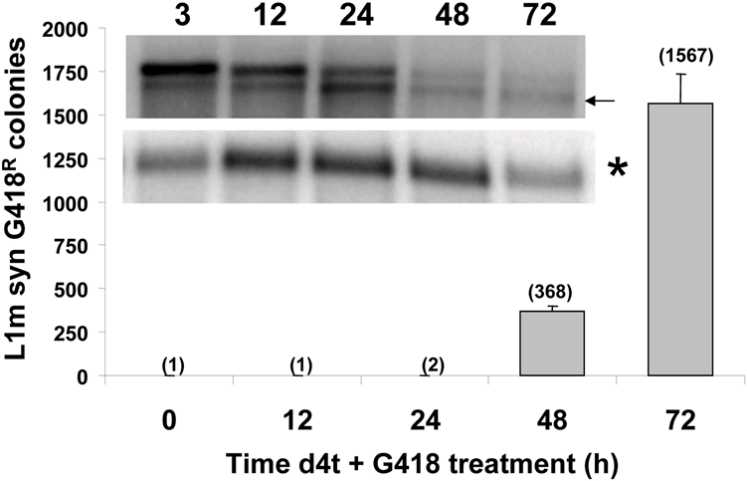
L1 Retrotransposition Rate Does Not Correlate with the Timing of Insertion. The retrotransposition capability of the L1 construct with the highest retrotransposition rate reported (L1m syn) [52] was evaluated. HeLa cells were transiently transfected and treated with d4t plus G418 at different time points post-transfection or harvested for RNA extractions. Bars represent the mean G418^R^ colonies±standard deviation. The total number of G418 resistant colonies of the early time points is shown in parentheses. The top panel shows the northern blot analysis of the tagged L1m syn transcripts with the time points indicated above, where the arrow indicates the spliced product. The lower panel shows the β-actin transcripts (*).

Recent data demonstrate that one amino acid substitution in the mouse L1 ORF1 protein dramatically affects retrotransposition rate and the ability to detect new inserts earlier [50]. We evaluated the insertion timing of the most efficient L1 available at the time, the synthetic mouse L1 (L1m syn) previously reported to increase retrotransposition efficiency by more than 200 fold relative to the wildtype L1spa element [52]. Despite having a much higher retrotransposition rate, L1m syn required 48 h to generate inserts even when spliced RNA could be detected as early as 3 hours post-transfection (Figure 7). There were a few (1 to 2) colonies at 24 hours or earlier but these are likely outlier observations as they only represent 0.001 of the total observed G418^R^ colonies. Our data are consistent with the observation that all of the evaluated pol II-driven constructs require 48 h, while all of the pol III-driven constructs generate inserts by 24 h despite their very low retrotransposition rates relative to L1 (Table 1). Because of the large variation in retrotransposition rates, we opted to show the relative number of inserts in the figures for each construct by designating the 48 or 72 hour time point as 100%. While both U6 and *Alu* tagged transcripts, for example, can generate inserts by 24 hours, their retrotransposition rates (*i.e.*, the actual number of observed inserts) differ dramatically. The same is true for the tagged L1 and ORF1 RNAs.

**Table 1:** Relative retrotransposition rate of the different tagged constructs in HeLa cells under the same transfection conditions.

Mobile element	Rate G418^R^ colonies (×10^6^/µg at 72 h) mean±SD†	“Relative” rate*	Time requirement for insert (h)
L1¥	7880.0±321.0	5000	∼48
*Alu* (pol III)$	1053.3±160.8	500	∼24
7SL$	66.7±12.6	25	∼24
U6$	67.1±11.3	25	∼24
hY1$	16.7±1.6	10	∼24
hY3$	36.9±7.0	20	∼24
hY4$	57.5±6.4	25	∼24
hY5$	47.5±9.4	25	∼24
ORF1$ (pol II)	23.1±5.5	10	∼48
*Alu* (pol II)^	1.3±0.9	1	∼48

**†:** Rates were calculated by determining the number of G418 resistant colonies generated at 72 h, per 1 million cells per µg of transfected plasmid. Rates should not be considered as absolute numbers, as results will vary for different cell lines and conditions.

*: Due to the intrinsic experimental variation, a rough approximation was used to determine the relative retrotransposition rates. The lowest observed rate was arbitrarily designated as “1.”

**¥:** Data from the JM101/L1.3 construct.

$ Retrotransposition of the element was driven by an optimized L1 ORF2 expression plasmid (pBudORF2opt). Rates were much lower when a full-length wild type L1 was used as the driver for retrotransposition (not shown).

^ Retrotransposition of the element was driven by cotransfection with L1 ORF1 and ORF2 expression plasmids (pBudORF1opt and pBudORF2opt).

## Discussion

Throughout mammalian evolution different mobile elements have flourished within genomes. Retroelements such as LINEs and SINEs have been particularly successful, generating more than one third of human sequence mass. Interestingly, the parasitic non-autonomous SINE elements outnumber their autonomous LINE partners in the primate and rodent genomes. The success of SINEs is especially evident when compared to the copy numbers of other non-autonomous elements such as the retropseudogenes.

Our data reveal differences between retropseudogenes, *Alu*, and L1 retrotransposition. When evaluating *Alu* and L1 retrotransposition kinetics, the tagged *Alu* transcript required less time to generate an insert. This timing difference can not be attributed to differences in the time required to generate functional transcripts or availability of L1 proteins. It is clear that full-length functional L1 transcripts can be detected as early as 3 hours post-transfection and are abundant by 24 h post-transfection. In addition, the difference observed between *Alu* and L1 kinetics could not be attributed to the type of detection cassette system (self splicing or not) or to the differences in the retrotransposition rates. L1 colonies were rarely observed (Figure 7) at time points earlier than 48 h. These few observed G418^R^ colonies possibly represent the rare event that circumvented inhibition by d4t (in one experiment a colony was observed even at the zero time point). In our assay, production of L1 ORF2p is not limiting. Our data demonstrate that enough ORF2p is generated from an L1 construct to drive *Alu* insertions within 24 hours post-transfection, which indicates that ORF2p is made and readily available for *Alu* transcript mobilization. However, at this time we do not know if the ORF2p reaches the nucleus as a “free” protein or as part of an RNP with the L1 RNA or *Alu* RNA. As expected, due to the L1 *cis*-preference [10], pre-transfections with ORF1p, ORF2p or other L1 components, such as full-length transcripts or RNPs, did not affect the L1 time requirement.

Although unexpected, it is not totally surprising that *Alu* and L1 present different retrotransposition time requirements. Previous data show that, although *Alu* and L1 share the same insertion hallmarks, the two elements can exhibit differences in their behavior. For example, of two HeLa “cell lines,” only one supports *Alu* retrotransposition while both support L1 retrotransposition [62]. In addition, *Alu* and L1 are selectively inhibited by different APOBEC3 proteins [62]. This corroborates our observations that cellular components differentiate between Alu and L1 retrotransposition.

Our data suggest that the observed time differences are dependent on the type of RNA polymerase generating the transcript. Multiple features that distinguish these two transcript types may collectively or individually contribute to the observed differences in the retrotransposition timing between L1 and *Alu* elements. RNA capping, association with the translational machinery and ORF1 requirement are plausible factors that could influence SINE and LINE retrotransposition kinetics. As a pol II product, L1 mRNA is likely capped. Experimental evidence indicates that at least part of the L1 mRNA is capped [43] and that capping enhances L1 translation *in vitro* [44]. In contrast, pol III genes lack the 7-methylguanosine cap and are subjected to different processing in a spatially separate location of the nucleus [57],[58]. L1 mRNA likely interacts with most, if not all, of the pol II protein complexes that assemble with the transcription of generic mRNAs, as evidenced by the premature polyadenylation and splicing of L1 transcripts [6],[42].

Even though both pol II and pol III produced RNAs form complexes with various cellular proteins, the structure and composition of these RNPs varies dramatically. As a rule, pol III transcripts do not code for proteins and therefore interact with the translational machinery in a different manner than mRNA. Most known pol III transcripts fold to form a structured RNA and associate with a variety of proteins to form RNPs. Specifically, *Alu* interaction with SRP9 and SRP14 [12] is thought to transiently provide proximity to the ribosomal complexes and translating L1 RNA, allowing the *Alu* transcript to efficiently compete for the L1 factors required for retrotransposition [26]. It is also likely that the ability of the dimeric *Alu* to bind these proteins contributes to the dramatic difference in retrotransposition rates observed between *Alu* and other SINEs [9],[13]. In contrast, the polyribosomes and translation machinery assemble with the L1 mRNA in a more stable complex to undergo translation. The *cis*-preference displayed by L1 [20] suggests that the L1 RNA must dissociate from the cellular translation machinery to form L1 RNPs as an intermediate step in the retrotranspositional process. These L1 complexes are composed of L1 RNA, ORF1p [20], and likely ORF2 protein [11]. All three components are shown to co-purify in the polyribosomal fraction of the cytoplasm [11],[23]. It is plausible that ORF1p directly competes with the cellular translation machinery for access to L1 mRNAs, transitioning the L1 transcript away from the polyribosomal fraction and into the retrotranspositionally competent RNPs. Because of their nature and subcellular localization, SINEs completely avoid these two potentially time consuming steps in their mobilization. Therefore, SINE transcripts may enter their retrotransposition cycle as soon as L1 ORF2p becomes available.

The pol II-driven *Alu* transcripts that are most likely to associate with the cellular translational machinery, at least transiently, require L1 ORF1 protein in addition to ORF2 protein for retrotransposition in a manner reminiscent of retropseudogenes [29]. The retrotransposition time of the pol II-driven *Alu* parallels that of L1. At this stage it is unclear what the role of ORF1p is in the *trans*-mobilization of retropseudogenes or the pol II *Alu* transcript. However, it is consistent with the above-discussed hypothesis implicating ORF1 protein in removing pol II RNAs from their expected cycle of translation and degradation. Thus, the pol II L1 and the pol III *Alu* transcript interactions with different cellular components may dictate the timing difference between L1 and *Alu* RNAs to form their respective retrotranspositionally competent complexes.

The inefficient retrotransposition rate of the pol II-driven *Alu* construct suggests that the presence of an *Alu* sequence within an mRNA would not facilitate its retrotransposition by L1 factors. Although there is no available data on the SVA promoter, it is unlikely that the pol III polymerase drives SVA transcription due to the presence of numerous pol III terminators within its sequence. Thus, it is questionable whether the truncated antisense *Alu*-like sequences present in the SVA element contribute to the L1 *trans*-complementation of this retroposon as previously suggested [35].

In addition to assisting its own retrotransposition, the *cis*-preference exhibited by L1 may decrease cell damage by limiting random retrotransposition of cellular mRNA. A previous study demonstrated the co-localization of ORF1p and cellular proteins to stress granules[63]. The authors suggest that the sequestering of ORF1 protein in stress granules for degradation may prevent promiscuous binding of ORF1p to non-L1 mRNAs. Thus, as a side effect of L1 self-preference, retropseudogene formation is less likely [5]. In addition, this “*cis*-preference” could help the L1 transcript “escape” the ribosomal complex and degradation pathways. Once translation is completed, most transcripts decay by several known mRNA degradation pathways, reviewed in [56]. In contrast, pol III transcripts are meant to perform their function as RNA molecules in the cytoplasm or nucleus before degradation by the exosome [64]. Essentially, the functional molecule of pol III genes is the RNA, while for pol II genes the mRNA is an intermediary prior to the generation of the functional protein. In the case of L1, the ORF1p may play an additional role by protecting the L1 RNA from degradation, increasing the chance of returning to the nucleus where the involvement of ORF1p in the L1 integration process has been previously suggested [18],[23]. Thus, the requirement for both ORF1 and ORF2 proteins could contribute to the longer time needed for L1 transcripts to generate inserts. In addition, it is plausible that interactions with different cellular components during insertion, mediated by ORF1p, may contribute to the timing differences observed.

**Figure 8:**
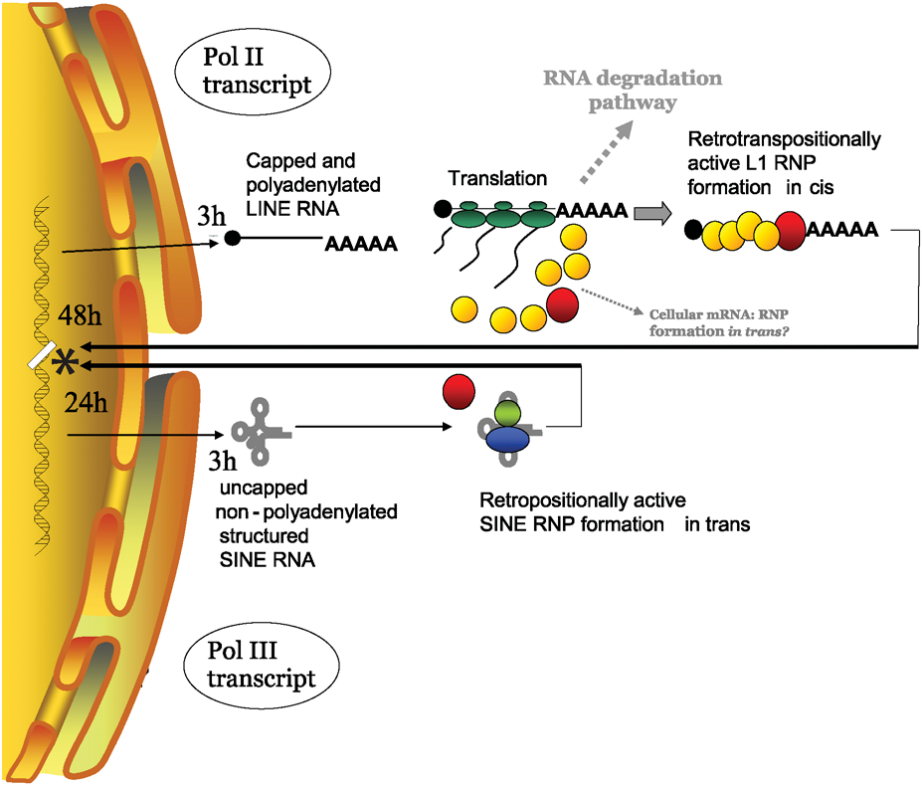
Model of SINE and LINE Cellular Interactions Potentially Contributing to Differences in Retrotransposition Kinetics. We present a model of how pol II (L1 and mRNA) and pol III transcript interactions with their respective cellular components in the cytoplasm may influence the retrotransposition timing of LINEs and SINEs. Structures are not drawn to scale. Transcription and processing are not the limiting steps for L1 and Alu, as both pol II and pol III spliced tagged transcripts are present in the cytoplasm as early as 3 h post-transfection. However, Alu requires about 24 h to generate an insert, while L1 requires about 48 h (*). Pol II transcript: LINE RNA and pol II-driven mRNAs reach the cytoplasm after processing and modifications. The cytoplasmic pol II transcript has been spliced, polyadenylated and capped at its 5′ end (shown as a black dot at the end of the RNA). The cap allows the recognition by several proteins involved in translation forming a large protein complex interacting with the transcript. Capping also allows for the association with the PABP-1, elongation factors and the circularization of the mRNA (not shown). This large multi-protein complex interacts with the translation machinery to generate the needed ORF1 and ORF2 proteins. The generated proteins will preferentially bind to the L1 RNA that encoded them (*cis*-preference). Multiple ORF1p molecules (yellow circles) and possibly ORF2p (red circle) bind the L1 transcript. We propose that the formation of the L1 RNP complex will allow the L1 RNA to separate from the translation machinery and evade the normal degradation pathway en route to the nucleus. This process of detachment from the ribosomal complex and avoidance of the RNA decay pathways may increase the time requirement for L1 retrotransposition. These extra steps probably contribute to the extended time requirement for L1 to complete the retrotransposition process. The L1 RNA (likely as an RNP with ORF1 and ORF2) reaches the nucleus and generates a new insert (represented as a white box in the DNA). In our assay system, the insertion process of L1 elements requires about 48 hours for completion. Cellular RNAs (*e.g.*, mRNAs and the pol II *Alu* RNA) can occasionally use the L1 proteins to mediate their mobility in *trans*. However, retropseudogene (pol II mRNA) inserts are not efficiently generated in our experimental system and require ORF1p. We propose that the spurious interaction with ORF1p allows these mRNAs to be shunted to the nucleus to go through the retrotransposition process. In addition, it is likely that ORF1p also contributes to the retrotransposition process in the nucleus. The ORF1*mneo* transcript generates inserts more efficiently than the other tested pol II transcripts, possibly because of close proximity to ORF1p in *cis*. Overall, the efficiency of a pol II transcript to generate inserts in tissue culture is likely correlated with its ability to interact with ORF1p. The role of ORF2p in the cytoplasm is unclear. Pol III transcript: SINE RNA reaches the cytoplasm with little or no processing and interacts with specific proteins. The cytoplasmic SINE RNP is stable and compact. In the case of *Alu*, the transcript forms a specific structure which binds the SRP9 (green circle) and SRP14 (blue circle) proteins. It is hypothesized that these proteins may target SINE RNA to the ribosomes, generating transient close proximity to nascent L1 proteins that might be essential for SINEs to efficiently use L1 in *trans* for retrotransposition. Although SINE RNPs may be targeted to the ribosomes, they are not functional components of the translational complex, making this interaction likely transitory. Whether pol III RNA gains access to the L1 retrotransposition machinery in the cytoplasm or in the nucleus remains undetermined. Because SINE transcripts are not translated or functional components of the translational complex, the SINE RNPs are likely “free” to sequester the L1 proteins and immediately proceed with the retrotransposition cycle. We propose that the pol III SINE probably reaches the nucleus in a more efficient manner than the pol II transcripts, such as the L1 RNA, which must first dissociate from the translational complex and avoid the normal mRNA degradation pathway. In our system, the insertion process of an Alu requires 24 hours or less for completion (* represented as a white box in the DNA).

We postulate that the differences observed in retrotransposition kinetics are dictated by the type of RNA polymerase generating the transcript. We propose an initial model where the cytoplasmic interactions of pol II (L1 and mRNA) and pol III transcripts and pathways influence the amplification kinetics of LINEs and SINEs (Figure 8). Overall, it is evident that the type of RNA polymerase generating the transcript alters the timing of mobile element insertion and remains a critical parameter in the classification of different types of retroelements.

## Materials and Methods

### Plasmids

#### L1 related vectors

JM101/L1.3 referred to “L1*mneo*” contains a full-length copy of the L1.3 element and the *mneo*I indicator cassette cloned in pCEP4 (Invitrogen) [61],[65].

JM101/L1.3 no tag, referred as “L1 no tag” contains a full-length copy of the L1.3 element cloned in pCEP4 [10].

TAM102/L1.3 contains the full-length copy of L1.3 element and the *mblastI* indicator cassette cloned in pCEP4 [32].

ORF1*mneo* contains the L1.3 5′UTR, L1.3 ORF1, and the *mneo*I indicator cassette cloned in pCEP4 [10].

psmL1 contains the codon optimized L1spa sequence and the neomycin indicator cassette cloned in pCEP4 [52].

L1*neo* ^TET^ contains the codon optimized L1_RP_ driven by CMV promoter and tagged with the self splicing intron neo cassette [4] in pBluescript.

JM101/L1.3, JM101/L1.3 no tag, TAM102/L1.3 and ORF1*mneo* were kind gifts from Dr. John Moran. psmL1 was a kind gift from Dr. Jef Boeke [52]. pBudORF2opt [33]and pBudORF1opt [66] have been previously described. The open reading frames are cloned into the expression vector pBudCE4.1 (Invitrogen), under control of the CMV promoter.

#### SINE related vectors

Alu-*neo* ^TET^ containing a 7SL upstream enhancer region - Alu core sequence followed by the *neo* ^TET^ self-splicing indicator cassette and 44 A-stretch followed by a pol III terminator [4] was a kind gift from Dr. Thierry Heidmann.

The “SINE”*neo* ^TET^ constructs listed below were created by initially modifying the Alu*neo* ^TET^ vector. The QuickChange site-directed mutagenesis kit (Stratagene) was used to introduce an *Aat*II site (underlined) at the 3′ end of the *Alu* element with a set of complementary 5′ phosphorylated primers to the following sequence: 5′- AGCCTGGGCGACAGAGCGAGTCGACGTCTCAAATCCCCTCAG -3′ following the manufacturer's recommended protocol. The new construct, referred to as AluYa5*neo* ^TET^ *Aat*II, was then used to introduce the different individual elements and their corresponding upstream enhancer sequences using the *BamH*I (5′ of 7SL promoter enhancer sequence) and the *Aat*II sites (schematic of the basic vector shown in Figure 2A). The *BamH*I and *Aat*II sites are underlined.

AluYa5*neo* ^TET^, contains a larger amount of the upstream pol III enhancer sequence of the 7SL gene (113 bp) and the *Alu*Ya5 consensus sequence from p^7SL^Ya5^BC1^ [67].

7SL*neo* ^TET^, contains a 413 bp fragment of the Human 7SL RNA gene Accession # M20910 and its upstream pol III enhancer sequence, PCR amplified with primers F7SL: 5′-GGATCCGCCCAGTGTGGGTGTGTCC-3′ and R7SL: 5′ GACGTCAAGAGACGGGGTCTCGCTATG-3′

U6*neo* ^TET^, contains a 512 bp fragment of the human small nuclear RNA gene HUMUG6 Accession# M14486 and its upstream pol III enhancer sequence PCR amplified with primers FU6: 5′-GGATCCGCAGACACTGCTCGGTAGTT-3′ and RU6: 5′ GACGTCAAGAGACGGGGTCTCGCTATG-3′

hY1*neo* ^TET^, contains a 478 bp fragment of the human RNA gene hY1 encoding Ro RNA Accession# V00584 and its upstream pol III enhancer sequence PCR amplified with primers FhY1: 5′-GGATCCGTCCCACAGAGCTGTCCGGAGG-3′ and RhY1: 5′-GACGTCAAGACTAGTCAAGTGCAGTAGTGAG-3′

hY3*neo* ^TET^, contains a 430 bp fragment of the human RNA gene hY3 encoding Ro RNA Accession# V00585 and its upstream pol III enhancer sequence PCR amplified with primers FhY3: 5′-GGATCCCGCTCTAGACGTCCTGGCC-3′ and RhY3: 5′-GACGTCAAGGCTAGTCAAGTGAAGCAG-3′

hY4*neo* ^TET^, contains a 494 bp fragment of the human RNA gene hY4 encoding Ro RNA Accession# L32608 and its upstream pol III enhancer sequence PCR amplified with primers FhY4: 5′-GGATCCACAGGCAGGGAFACGACAAA-3′ and RhY4: 5′-GACGTCAAGCCAGTCAAATTTAGCAGTGG-3′

hY5*neo* ^TET^, contains a 667 bp fragment of the human RNA gene hY5 encoding Ro RNA Accession# K01564 and its upstream pol III enhancer sequence PCR amplified with primers FhY5: 5′-GGATCCTGATGATGAAACAAAGCC-3′ and RhY5: 5′-GACGTCAACAGCAAGCTAGTCAAGC-3′.

B2*neo* ^TET^, contains 113 bp of the upstream pol III enhancer sequence of the 7SL gene and the B2 sequence from p^7SL^B2^BC1^ [67].

^CMV^Ya5*mneo* (“pol II *Alu*”) the *mneo*I cassette including the SV40 polyadenylation signal from JM101/L1.3 was amplified by PCR with the high fidelity phusion DNA polymerase (New England Biolabs) using primers sets to introduce the 5′ *Fse*I and a 3′ *Bgl*II used for introduction into the compatible *Fse*I-*BamH*I sites in pGL3^CMV^Ya5^BC1-SV40pA^ [68]. The construct was then modified to eliminate the internal polyA-stretch immediately downstream of the *Alu* sequence and 5′ to the selection cassette (Figure 6). The pol II *Alu* transcripts generated from this construct are polyadenylated from the SV40pA signal.

pIRES2-EGFP (BD Biosciences Clontech) was used as the G418^R^ expression plasmid for toxicity control.

All plasmid DNA was purified by alkaline lysis and twice purified by cesium chloride buoyant density centrifugation. DNA quality was also evaluated by the visual assessment of ethidium bromide stained agarose gel electrophoresed aliquots. All new constructs were sequence verified

### Retrotransposition Assays

The basic transient L1 [16] or *Alu* [4] retrotransposition assay was performed as previously described with some minor modifications. Briefly, HeLa cells (ATCC CCL2) were seeded in T-75 flasks at a density of 5×10^5^ cells/flask or in 6 well plates at a density of 2.5–5×10^4^/well. Transient transfections were performed the next day with Lipofectamine Plus following the manufacturer's protocol (Invitrogen), with 3 µg of SINE-*neo* ^TET^ vector plus 1 µg pBud-ORF2opt or 1 µg of L1 no tag. For L1 assays 1 µg of JM101/L1.3 was used. Inhibitory effects on cellular growth or colony formation capabilities by the d4t treatment was evaluated by transfecting cells in parallel with 0.3 µg of a plasmid expressing neomycin resistance (pIRES2-EGFP; BD Biosciences Clontech) as a “toxicity” control. Following removal of transfection cocktail, the cells were treated with the appropriate media containing 400 µg/ml Geneticin/G418 (Fisher Scientific) alone or in combination with 50 µM d4t for selection and/or reverse transcriptase inhibition. After 14 days, cells were fixed and stained for 30 minutes with crystal violet (0.2% crystal violet in 5% acetic acid and 2.5% isopropanol). The inhibitor d4t- (2′,3′-Didehydro-3′-deoxy-thymidine; Sigma-Aldrich) was freshly added to the selection media at the indicated time period. During the inhibitor treatment period all cells in the experiment were refreshed daily for the first week with the appropriate media. The rate of insertion efficiency (retrotransposition rate) was determined as the number of visible G418^R^-resistant colonies obtained at 72 h after transient transfection of 1×10^6^ seeded HeLa cells with 1 µg of the neo tagged construct.

### Northern Blot Analysis

RNA extraction and poly(A) selection was performed as previously described [42]. Total RNA was extracted using the recommended protocol for TRIzol Reagent (Invitrogen) from two 75 cm^2^ cell culture flasks at 3, 6, 24, 48, and 72 hours post-transfection. The PolyATract mRNA isolation system III (Promega) was used to select polyadenylated RNA species following the manufacturer's protocol. After separation in a 1% (L1) or a 2% (pol III constructs) agarose-formaldehyde gel, the RNA was transferred to a Hybond-N nylon membrane (Amersham Biosciences). The RNA was cross-linked to the membrane using a UV-light (GS Gene linker, BioRad) and pre-hybridized in 30% formamide, 1× Denhardt's solution, 1% SDS, 1 M NaCl, 100 µg/ml salmon sperm DNA, 100 µg/ml-1 yeast t-RNA at 60°C for at least 6 h. The 3′ region of the neomycin gene was amplified by PCR using the following primers T7neo (−): 5′-TAATACGACTCACTATAAGGACGAGGCAGCG-3′ and Neo northern (+): 5″- GAAGAACTCGTCAAGAAGG-3′. The isolated PCR product was used as a DNA template to generate a ^32^P-CTP (Amersham Biosciences) labeled single strand-specific RNA probe using the MAXIscript T7 kit (Ambion) following the manufacturer's recommended protocol. We utilized material included in the kit to generate the riboprobe for the β-actin. The radiolabeled probe was purified by filtration through a NucAway Spin column (Ambion). Hybridization with the probe (final concentration of 4–12×10^6^ cpm/ml) was carried out overnight in the pre-hybridization solution at 60°C. Two ten-minute washes were performed at high stringency (0.1×SSC, 0.1%SDS) at 60°C. The results of the northern blot assays were evaluated using a Typhoon Phosphorimager (Amersham Biosciences) and the ImageQuant software.

## Supporting Information

Figure S1 Evaluation of D4t Inhibitory Concentration 50 (IC_50_) on L1 and *Alu* Retrotransposition. HeLa cells were transiently transfected with plasmids expressing a neomycin-tagged L1 (solid line) or a marked Alu supplemented with an ORF2p expression vector (dashed line). Cells were treated with different concentrations of d4t and G418 for two weeks. Colonies were stained and scored. The no treatment data were used to define 100%. The relative % mean G418^R^ colonies±standard deviation are shown for each construct. The inhibitory concentration 50 (IC_50_) for L1 and Alu is ∼2 µM d4t, the intercept (gray line) is shown.
(0.34 MB TIF)

Figure S2 The earliest detection of L1 inserts occurred at 32 hours post-transfection. HeLa cells were transiently transfected with plasmids expressing a neomycin-marked L1 (black), marked Alu supplemented with an ORF2p expression vector (gray) or a control vector with neomycin resistance (white). Cells were treated with G418 plus d4t at 24, 32, 42, 48 and 72 h post-transfection (x axis). The 72 h data were used to define 100%. Bars represent the relative % mean G418^R^ colonies±standard deviation shown as error bars for each construct.
(0.44 MB TIF)

Figure S3 Vector transcription kinetics. Cells were transiently transfected with 5 µg of the tagged vectors. RNA was extracted at different time points (3–72 h) after transfection. Poly-A selected transcripts were evaluated by Northern blot analysis using an RNA strand specific probe to the neomycin resistance gene or to β-actin (indicated by an *). RNA is transcribed as early as 3 hours post-transfection. L1*mneo*, AluYa*5neo* ^TET^ and L1*neo* ^TET^ are shown. Transcripts containing the unspliced (open arrowhead) and spliced (black arrow) neo indicator cassette are indicated. Only the spliced transcripts are able to generate inserts conferring G418 resistance and these transcripts were used for the RNA quantitation. Exposures times varied due to the strong signal from the later time periods.
(2.92 MB TIF)

Figure S4 Supplementation with L1 factors does not affect L1 insertion kinetics. The schematic of transfection and treatment timeline is shown. Cells were stained after 2 weeks of treatment. To ensure the early presence of the L1 factors, HeLa cells were pre-transfected (T1) with plasmids expressing L1 ORF1p, ORF2p ORF1p plus ORF2p, and a untagged L1 (as a source of L1 RNA and or RNPs) 24 h before introducing the tagged L1 element (T2). Cells were treated with d4t and G418 at 24, 48, 72 and 96 h post-transfection (x axis). The 96 h data were used to define 100%. Bars represent the relative % mean G418^R^ colonies±standard deviation shown as error bars for each construct.
(0.54 MB TIF)

Table S1 Evaluation of splicing efficiency of the tagged L1 and Alu constructs at different time points.
(0.05 MB DOC)
