# Supplementary figures and images for: The RNA Polymerase Dictates ORF1 Requirement and Timing of LINE and SINE Retrotransposition
Source: PLoS Genet. 2009 Apr 24;5(4):e1000458. doi: 10.1371/journal.pgen.1000458 (PMC2666806; doi:10.1371/journal.pgen.1000458)

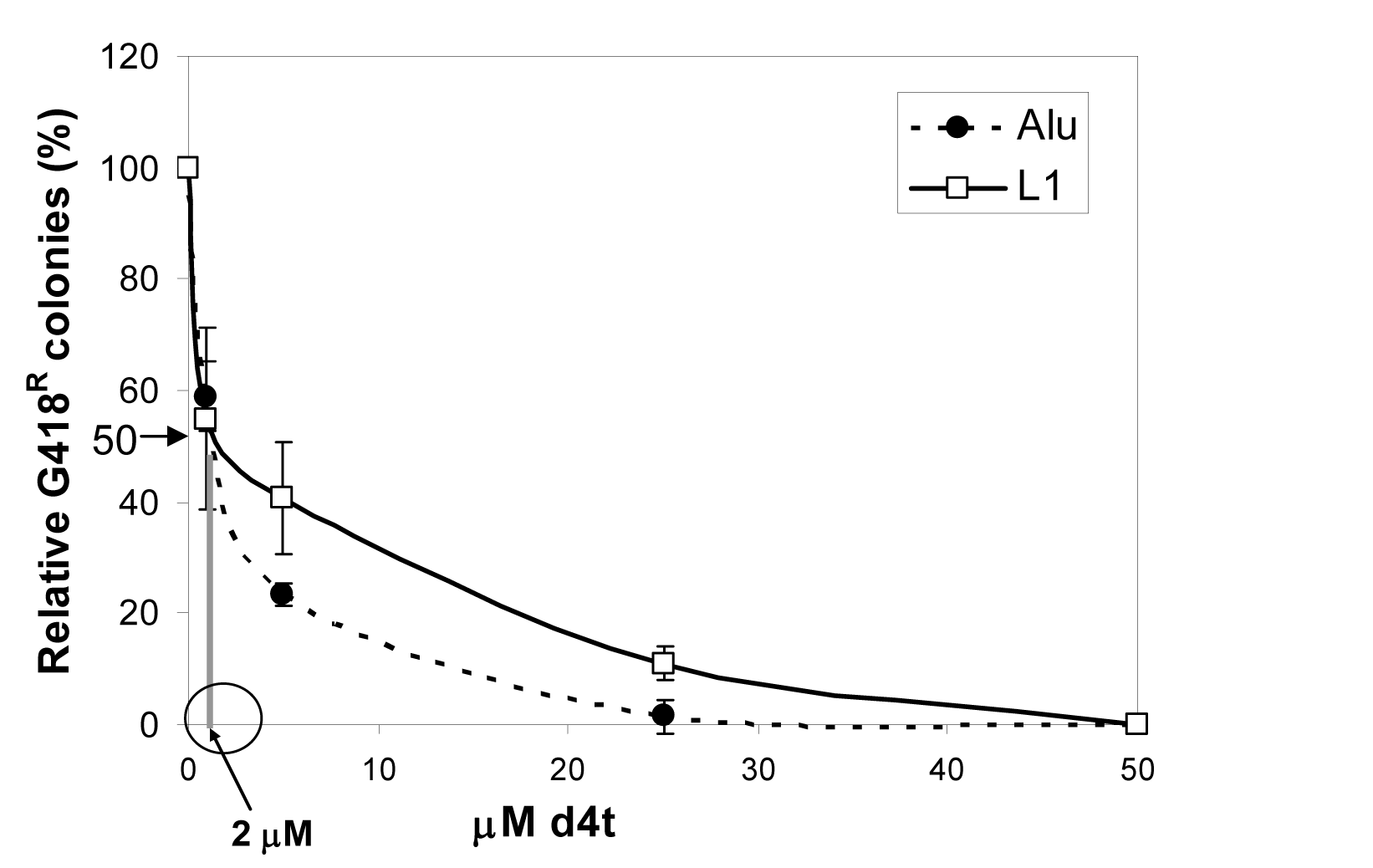

Supplement: Figure S1 — Evaluation of D4t Inhibitory Concentration 50 (IC50) on L1 and Alu Retrotransposition. HeLa cells were transiently transfected with plasmids expressing a neomycin-tagged L1 (solid line) or a marked Alu supplemented with an ORF2p expression vector (dashed line). Cells were treated with different concentrations of d4t and G418 for two weeks. Colonies were stained and scored. The no treatment data were used to define 100%. The relative % mean G418R colonies±standard deviation are shown for each construct. The inhibitory concentration 50 (IC50) for L1 and Alu is ∼2 µM d4t, the intercept (gray line) is shown. (0.34 MB TIF) [file pgen.1000458.s001.tif]

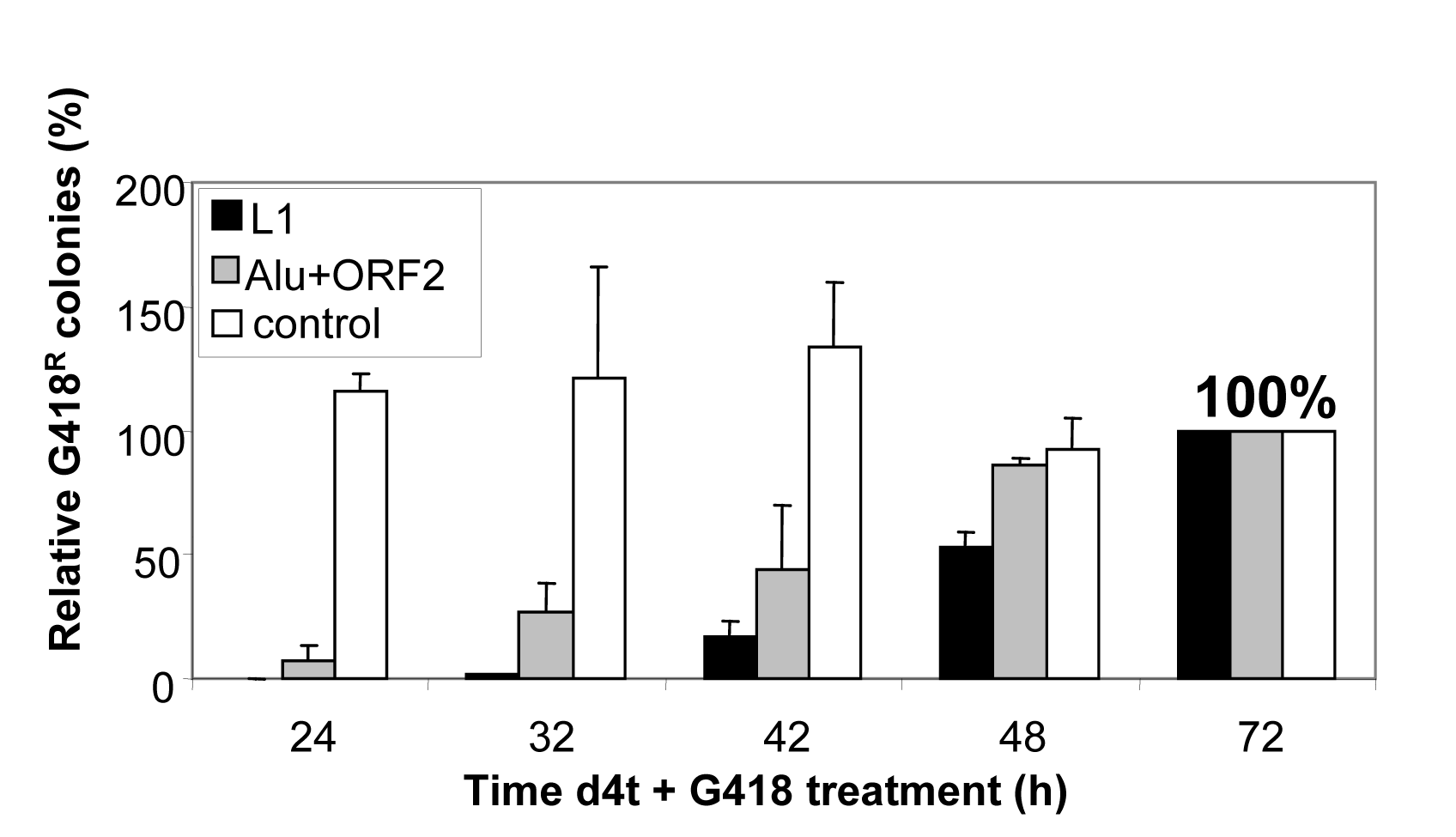

Supplement: Figure S2 — The earliest detection of L1 inserts occurred at 32 hours post-transfection. HeLa cells were transiently transfected with plasmids expressing a neomycin-marked L1 (black), marked Alu supplemented with an ORF2p expression vector (gray) or a control vector with neomycin resistance (white). Cells were treated with G418 plus d4t at 24, 32, 42, 48 and 72 h post-transfection (x axis). The 72 h data were used to define 100%. Bars represent the relative % mean G418R colonies±standard deviation shown as error bars for each construct. (0.44 MB TIF) [file pgen.1000458.s002.tif]

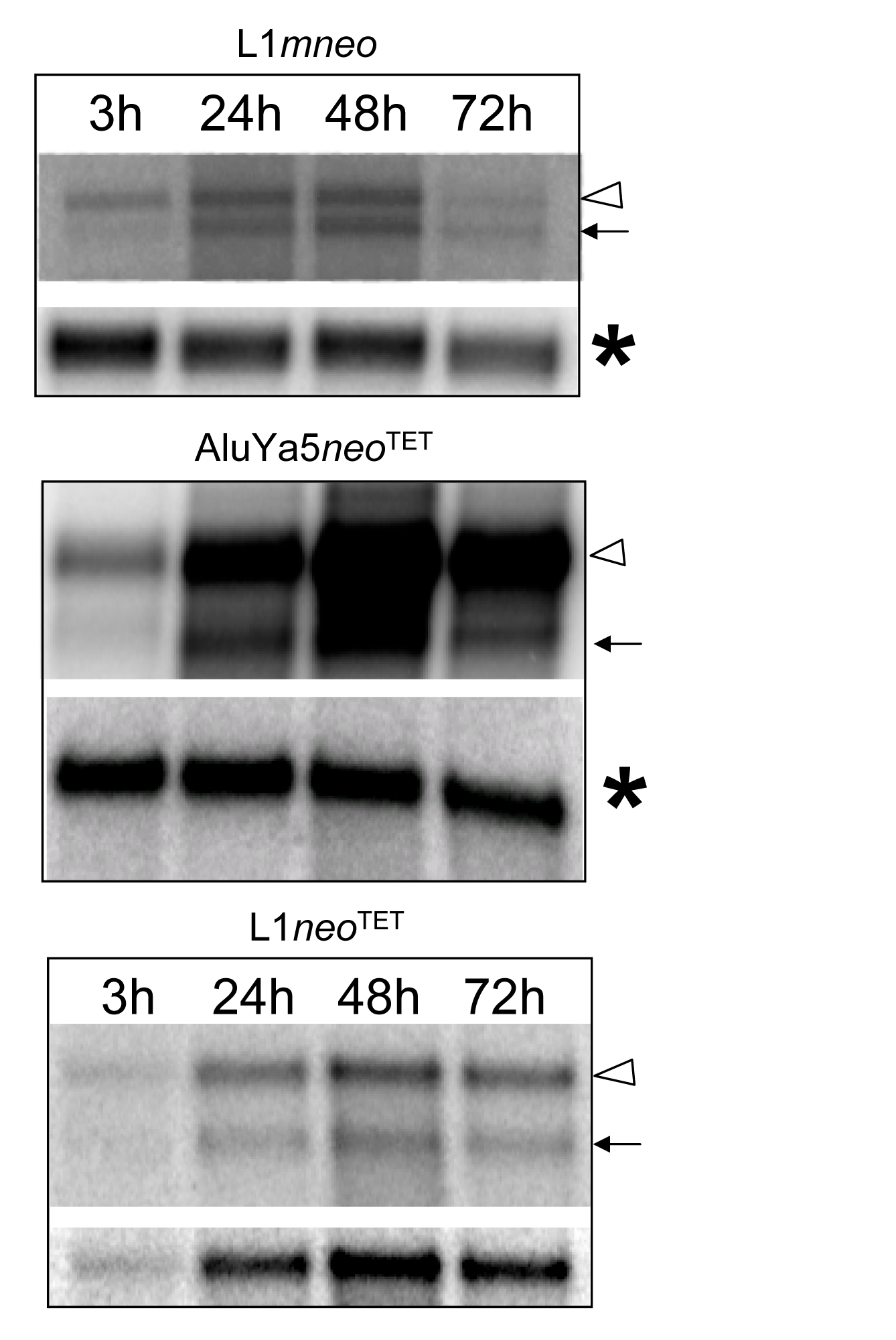

Supplement: Figure S3 — Vector transcription kinetics. Cells were transiently transfected with 5 µg of the tagged vectors. RNA was extracted at different time points (3–72 h) after transfection. Poly-A selected transcripts were evaluated by Northern blot analysis using an RNA strand specific probe to the neomycin resistance gene or to β-actin (indicated by an *). RNA is transcribed as early as 3 hours post-transfection. L1mneo, AluYa5neo TET and L1neo TET are shown. Transcripts containing the unspliced (open arrowhead) and spliced (black arrow) neo indicator cassette are indicated. Only the spliced transcripts are able to generate inserts conferring G418 resistance and these transcripts were used for the RNA quantitation. Exposures times varied due to the strong signal from the later time periods. (2.92 MB TIF) [file pgen.1000458.s003.tif]

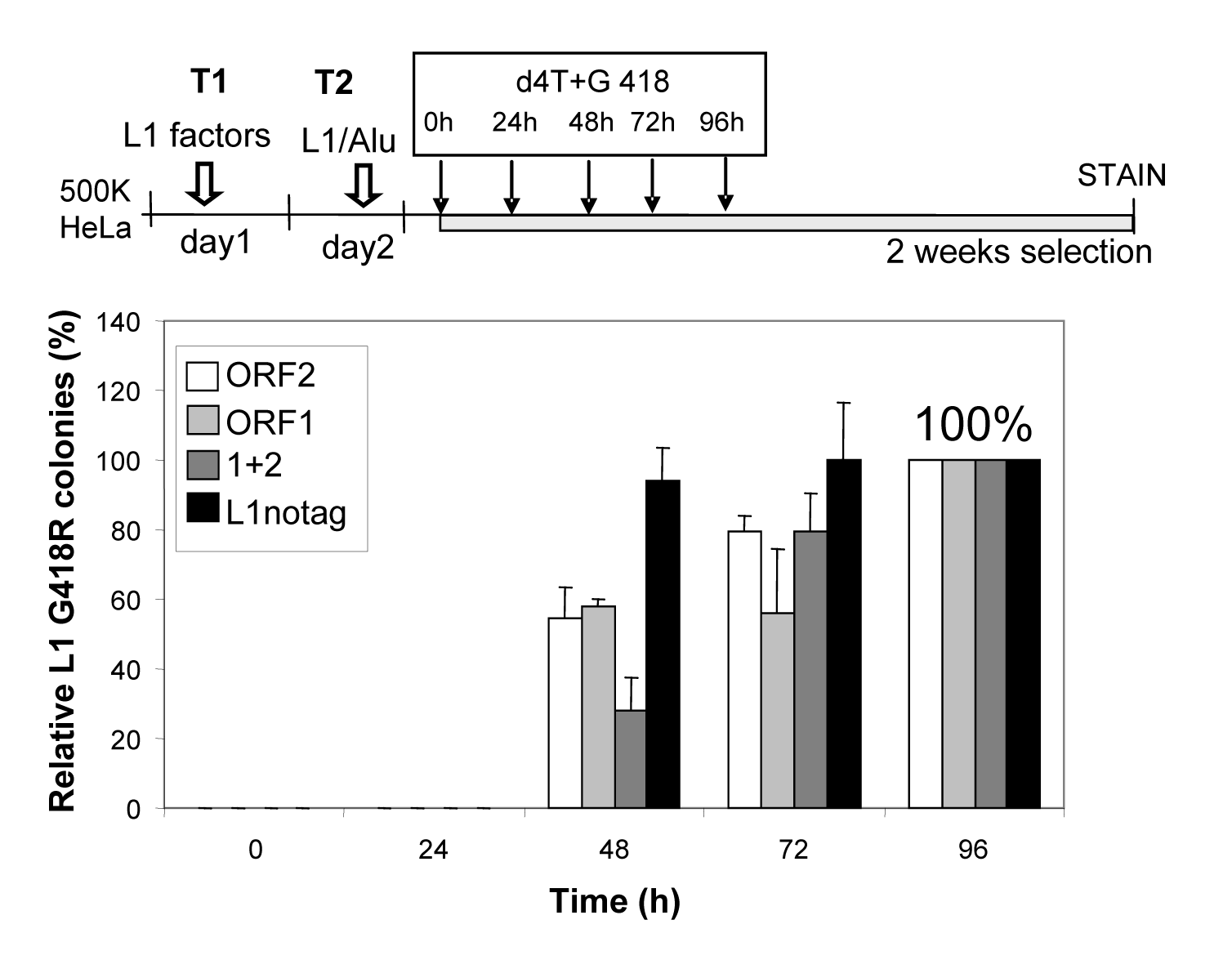

Supplement: Figure S4 — Supplementation with L1 factors does not affect L1 insertion kinetics. The schematic of transfection and treatment timeline is shown. Cells were stained after 2 weeks of treatment. To ensure the early presence of the L1 factors, HeLa cells were pre-transfected (T1) with plasmids expressing L1 ORF1p, ORF2p ORF1p plus ORF2p, and a untagged L1 (as a source of L1 RNA and or RNPs) 24 h before introducing the tagged L1 element (T2). Cells were treated with d4t and G418 at 24, 48, 72 and 96 h post-transfection (x axis). The 96 h data were used to define 100%. Bars represent the relative % mean G418R colonies±standard deviation shown as error bars for each construct. (0.54 MB TIF) [file pgen.1000458.s004.tif]
