# Supplementary material for: The RNA Polymerase Dictates ORF1 Requirement and Timing of LINE and SINE Retrotransposition
Source: PLoS Genet. 2009 Apr 24;5(4):e1000458. doi: 10.1371/journal.pgen.1000458 (PMC2666806; doi:10.1371/journal.pgen.1000458)
Supplement: Table S1 — Evaluation of splicing efficiency of the tagged L1 and Alu constructs at different time points. (0.05 MB DOC) [file pgen.1000458.s005.doc]

**Table S1.** Evaluation of splicing efficiency of the tagged L1 and Alu constructs at different time points.

|  | **% spliced transcript*** | | | |
| --- | --- | --- | --- | --- |
| **Time (h)** | **L1 (*mneo*)** | **L1 (*neo*^TET^)**  **Self-splicing** | **Alu (*neo*^TET^)**  **Self-splicing** | **Alu (*mneo*)**  **Pol II-Alu** |
| **3** | 26.5 + 4.6 **$** | 19.1 + 11.9 | 23.3 + 6.9 | 26.0 + 5.4 $ |
| **24** | 45.4 + 2.0 | 33.7 + 2.8 | 33.0 + 4.7 | 49.4 + 7.2 |
| **48** | 51.3 + 0.4 | 39.8 + 6.1 | 31.0 + 2.9 | 46.6 + 8.6 |
| **72** | 53.9. + 2.5 | 34.5 + 2.4 | 27.9 + 4.9 | 50.9 + 5.7 |

* Mean + S.D.= % ratio: spliced/ (spliced + unspliced).

No significant difference was observed in Alu*neo*^TET^ or L1*neo*^TET^ splicing efficiency between any of the time points evaluated.

$ transcription efficiency was only significantly different between 3h vs 24h, 48h or 72h (Student’s paired t-test p< 0.01)
